# The work of Pierre Magal on differential equations, functional analysis and mathematical biology

**DOI:** 10.1007/s00285-025-02273-2

**Published:** 2025-10-18

**Authors:** Jacques Demongeot, Thomas Hillen, Shigui Ruan, Glenn Webb

**Affiliations:** 1 Université Grenoble Alpes, Ageis, EA7407 France; 2 Department of Mathematical and Statistical Sciences, University of Alberta, Edmonton, AB T6G 2G1 Canada; 3 Department of Mathematics, University of Miami, Coral Gables, FL 33156 USA; 4 Mathematics Department, Vanderbilt University, Nashville, TN 37212 USA

**Keywords:** 34K05, 34K18, 34K20, 35K57, 35K90, 37C25, 37L05, 37L10, 39A10, 47D62, 47H20, 47J35, 92D15, 92D25, 92D30

## Abstract

Pierre Magal (1967–2024) was a leading researcher at the interface of differential equations, functional analysis, and mathematical biology. He made substantial contributions to both theoretical and applied aspects of these subjects. He published a dozen monographs, proceedings, and special issues and more than 125 journal articles. In this article we provide an introduction to Pierre’s contributions in some topics, including discrete population dynamics, integrated semigroup theory and abstract Cauchy problems with nondense domain, traveling waves in biological systems, uniform persistence and global attractors, cell-to-cell P-glycoprotein transfer in breast cancers, transfer problems in population dynamics and economics, and modeling of various epidemic problems, in particular his recent and extensive work on modeling COVID-19.

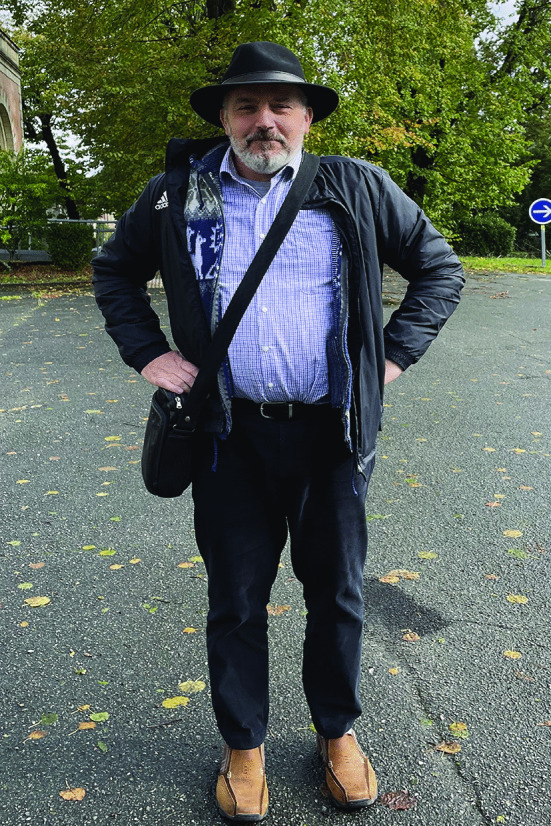



Pierre Magal in Bordeaux on November 7, 2023 (courtesy of Shigui Ruan)

*“Nature is written in mathematical language.”*–Galileo Galilei

*“Mathematics is the work of the human mind, which is destined rather to study*

*than to know, to seek the truth rather than to find it.”*–Évariste Galois

## Introduction

Pierre Magal was born on October 24, 1967, in La Rochelle, a coastal city in southwestern France and capital of the Charente-Maritime department. In 1993, Pierre received his Master Degree from University of Pau, France, and in 1996 he obtained his Ph.D. from the same university under the supervision of Ovide Arino (1947–2003). His thesis was titled *“Contribution à l’étude des systèmes dynamiques discrets préservant un cône et application à des modèles de dynamique de population”*. He did his Habilitation thesis, entitled *“Comportement asymptotique de modèles structurés en âges”*, at University of Le Havre, France, in 2003. From September 1997 to August 2009, he was an Associate Professor in Mathematics at University of Le Havre. In September 2009, Pierre moved to the University of Bordeaux, France, and was a Full Professor there till late December 2023, when he moved to Zhuhai, Guangdong Province, China. Unfortunately, while he was appointed as a Distinguished Professor at Beijing Normal University - Zhuhai for only one month, he become terminally ill in late January 2024. Four weeks later, Pierre passed away suddenly in Paris on February 20, 2024. Pierre is survived by his parents, a younger sister, former wife Christelle Suppo (who is also a mathematical biologist), son Enzo and daughter Clara.

Pierre was a leading researcher in the subjects of Differential Equations, Functional Analysis and Mathematical Biology. He possessed an extraordinary ability to study and seek understanding of these subjects and their applications. In this article we provide an introduction to Pierre’s contributions in some of the above mentioned subjects.

## Early research on discrete population dynamics

The first paper Pierre published was co-authored with D. Pelletier (Pelletier and Magal 1996) when he was still a graduate student. They studied the dynamics of a population that migrates seasonally in relation to reproduction. By using a two-patch two-season model, they compared population and catch levels obtained under different spatio-temporal allocations of fishing effort and characterized the dynamics of the saithe fishery in the west of Scotland in the 1980s. Their results showed that better biomass and catch levels could be attained by changing the allocation of fishing effort between seasons and patches, so that the effective age at entry in the fishery is optimized. Later, Pierre carried out theoretical analyses of such discrete population models systematically, such as on the existence (Magal and Pelletier 1997), uniqueness (Magal 1999) and global attractivity (Magal 2000, 2001b) of nontrivial equilibrium solutions for discrete population models and global asymptotic behavior of scalar delay difference equations (Magal 1998).

## Abstract Cauchy problems with nondense domain

In the 2001/2002 academic year, Shigui Ruan was on sabbatical at Vanderbilt University. In the fall term, Pierre was also visiting Vanderbilt. They both were working with Glenn Webb on various problems in mathematical biology, in particular age-structured biological models described by first order hyperbolic partial differential equations. Pierre and Shigui immediately found out that they have many common interests and started to collaborate, which turned out to be very productive.

### Integrated semigroups and Magal-Ruan operators

Recall that a linear operator $A:D(A)\subset X\rightarrow X$ on a Banach space $(X, \left\| .\right\| )$ is called a *Hille-Yosida operator* if there exist two constants, $\omega \in {\mathbb {R}}$ and $M\ge 1,$ such that $\left( \omega ,+\infty \right) \subset \rho \left( A\right) ,$ the resolvent set of *A*, and

$$\begin{aligned} \left\| \left( \lambda I-A\right) ^{-n}\right\| _{\mathcal {L}(X)}\le \frac{M}{\left( \lambda -\omega \right) ^{n}}, \; \forall \lambda >\omega , \; \forall n\ge 1, \end{aligned}$$

where $\Vert \;\Vert _{\mathcal {L}(X)}$ denotes the operator norm on $\mathcal {L}(X)$. The Hille-Yosida theorem (see Engel and Nagel 2000; Pazy 1983) says that a linear operator *A* is the infinitesimal generator of a strongly continuous semigroup $\left\{ T_A(t)\right\} _{t\ge 0}$ on a Banach space *X* if and only if (a) *A* is densely defined (i.e. $\overline{D(A)}=X$); (b) *A* is a Hille-Yosida operator. Thus, the Hille-Yosida theorem implies that the abstract Cauchy problem

3.1 $$\begin{aligned} \frac{dv(t)}{dt}=A v(t)+F(v(t)), \;\; t>0, \;\; v(0)=x \end{aligned}$$

is well-posed on *X* with respect to mild solutions whenever *A* is a densely defined Hille-Yosida operator and *F* is Lipschitz on *X*. In that case there is a unique mild solution for each $x \in X$ given by the variation of constant formula

3.2 $$\begin{aligned} v(t)=T_A(t) x + \int ^t_0 T_A(t-s)F(v(s))ds, \;\; t\ge 0. \end{aligned}$$

This fundamental result has many applications for biological models, in particular for models of reaction-diffusion type. However, this is not so for general age-structured models.

Consider an age-structured equation of the form

3.3 $$\begin{aligned} \begin{aligned}&\frac{\partial u(t,a)}{\partial t}+\frac{\partial u(t,a)}{\partial a}=-\mu u(t,a),\;\;t>0,\;a>0,\\&u(t,0)=f\left( \int _0^\infty \beta (a) u(t,a)da\right) ,\\&u(0,.)=u_0\in L^1_+(0,\infty ), \end{aligned} \end{aligned}$$

where *u*(*t*, *a*) denotes the population density at time *t* and age *a*, $\mu$ is a constant death rate, $\beta (a)$ is an age-dependent reproduction rate, and $f(\cdot )$ is a possibly nonlinear transformation from offspring into living individuals of age 0. In order to tackle the existence theory for (3.3), we rewrite it as an abstract Cauchy problem on a Banach space.

Define a Banach space $X={\mathbb {R}}\times L^1(0,\infty )$ and consider a linear operator $A:D(A)\subset X\rightarrow X$ defined by

$$\begin{aligned} D(A)=\{0\}\times W^{1,1}(0,\infty ),\;\; A\begin{pmatrix} 0\\ \varphi \end{pmatrix}=\begin{pmatrix} -\varphi (0)\\ -\varphi '-\mu \varphi \end{pmatrix},\;\;\forall \begin{pmatrix} 0\\ \varphi \end{pmatrix}\in D(A). \end{aligned}$$

By setting $$v(t)=\begin{pmatrix} 0\\ u(t,.)\end{pmatrix}$$, problem (3.3) can be reformulated in the form of equation (3.1) with $$x=\begin{pmatrix} 0\\ u_0\end{pmatrix}\in \{0\}\times L^1(0,\infty )$$ and $F:\{0\}\times L^1(0,\infty )\rightarrow X$ is the nonlinear map

$$ F\begin{pmatrix} 0\\ \varphi \end{pmatrix}=\begin{pmatrix} f\left( \int _0^\infty \beta (a) \varphi (a)da\right) \\ 0\end{pmatrix}. $$

Since $\overline{D(A)}=\{0\}\times L^1(0,\infty )\ne X$, the abstract Cauchy problem for the age-structured model (3.3) now has a nondensely defined linear operator *A*. Different types of equations with nondense domain had been studied by some researchers such as Ezzinbi and Adimy (2006), Da Prato and Sinestrari (1987), Thieme (1990a) and so on. Notice that in the case of $\overline{D(A)} \ne X$ the above variation of constants formula (3.2) may be not well-defined. However, it was observed that one may be able to integrate the equation twice to recover the well-posedness and the concept of an *integrated semigroup* was born.

#### Definition 3.1

(Thieme 1990b; Magal and Ruan 2018) Let $\left\{ S(t)\right\} _{t\ge 0}$ be a family of bounded linear operators on a Banach space $(X, \left\| .\right\| )$. We say that $\left\{ S(t)\right\} _{t\ge 0}$ is an *integrated semigroup* on *X* if the following properties are satisfied: (i) $S(0)=0;$ (ii) $t\rightarrow S(t)x$ is continuous from $\left[ 0,+\infty \right)$ into *X* for each $x\in X;$ (iii) *S*(*t*) satisfies

$$ S(t)S(s)=\int _{0}^{t}[S(r+s)-S(r)]dr, \; \forall t,s\in \left[ 0,+\infty \right) . $$

An integrated semigroup $\left\{ S(t)\right\} _{t\ge 0}$ is * nondegenerate* if

$$\begin{aligned} S(t)x=0, \forall t\ge 0 \Rightarrow x=0. \end{aligned}$$

A linear operator $A:D(A)\subset X\rightarrow X$ is said to be the *generator* of $\left\{ S(t)\right\} _{t\ge 0}$ if and only if

$$\begin{aligned} x\in D(A)\text { and }y=Ax\Leftrightarrow S(t)x=tx+\int _{0}^{t}S(s)yds, \forall t\ge 0. \end{aligned}$$

Integrated semigroups have been studied extensively by many researchers including Adimy (1983), Arendt (1987a, 1987b), Hieber (1991a, 1991b), Kellermann and Hieber (1989), Neubrander (1988), Thieme (1990b, 2008), and have been used to investigate various types of differential equations. It is known that $\left\{ S_A(t)\right\} _{t\ge 0}$ is a nondegenerate integrated semigroup and *A* is its generator if and only if there exist two constants $\omega >0$ and $M>0$ such that

$$\begin{aligned} \left( \omega ,+\infty \right) \subset \rho \left( A\right) , \;\; \left\| S_A(t)\right\| _{\mathcal {L}\left( X\right) }\le Me^{\omega t}, \; \forall t\ge 0, \end{aligned}$$

and

$$\begin{aligned} (\lambda I-A)^{-1}x=\lambda \int _{0}^{\infty }e^{-\lambda s}S_A(s)xds,\;\;\forall \lambda >\omega . \end{aligned}$$

In 2007, Magal and Ruan (2007) developed some techniques and results for integrated semigroups when the generator is not a Hille-Yosida operator and is nondensely defined, and investigated the existence of mild solutions for nondensely defined nonhomogeneous Cauchy problems. In 2009, they (Magal and Ruan 2009a) further studied the positivity of solutions to the semilinear problem, the Lipschitz perturbation of the problem, differentiability of the solutions with respect to the state variable, time differentiability of the solutions, and the stability of equilibria. The operator that they introduced to treat nonhomogeneous Cauchy problems with nondense domain is now named a *Magal-Ruan (MR) operator* in the literature (Chen 2020; Li et al. 2024) which has been applied to study other types of equations with nondense domain.

#### Definition 3.2

(Magal and Ruan 2007, 2009a; Chen 2020; Li et al. 2024) Let *A* be a closed linear operator in a Banach space *X* with $\overline{D(A)} \ne X$ and $A_0$ be the part of *A* in $X_0=\overline{D(A)}.$ *A* is a *Magal-Ruan operator* if

(i) $A_0$ generates a $C_0$-semigroup $\{T_{A_0}(t)\}_{t \ge 0}$ in $X_0;$ i.e., $A_0$ is a Hille-Yosida operator in $X_0,$ so that *A* generates an integrated semigroup $\{S_A(t)\}_{t \ge 0},$ where $S_A(t)x=\int ^t_0 T_{A_0}(\ell )x d\ell , \; \forall t\ge 0, \; \forall x \in X_0;$
(ii) There is a real number $\tau _0>0$ and a nondecreasing function $\delta : [0, \tau _0] \rightarrow [0, \infty )$ such that $\delta (t) \rightarrow 0$ as $t \rightarrow 0^+$ and for any $f \in C^1([0, \tau _0], X),$
  $$\begin{aligned} \left\| \frac{d}{dt} (S_A * f)(t)\right\| \le \delta (t) \sup _{s \in [0, t]} \Vert f(s)\Vert , \;\; \forall t \in [0, \tau _0], \end{aligned}$$
  where $$\frac{d}{dt} (S_A * f)(t)=\int ^t_0 T_{A_0}(t-s)f(s)ds.$$

For the nonhomogeneous abstract Cauchy problem (3.1) with nondense domain, if the linear operator *A* is a Magal-Ruan operator and $F: \overline{D(A)} \rightarrow X$ is continuous, then results in Magal and Ruan (2007), Magal and Ruan (2009a) show that there is a unique integrated solution for each $x \in X$ given by

3.4 $$\begin{aligned} v(t)=T_{A_0}(t) x + \frac{d}{dt} (S_A * F)(t), \;\; \forall t \in [0, \tau _0]. \end{aligned}$$

Formula (3.4) is a generalization of the variation of constants formula (3.2) to the nondensely defined nonhomogeneous Cauchy problem when *A* is not a Hille-Yosida operator, which is crucial in studying center manifold theory, normal form theory and Hopf bifurcation in abstract Cauchy problems.

### Center manifolds, normal forms and Hopf bifurcation

In the 1980 s, some researchers (Cushing 1983; Diekmann et al. 1986; Hastings 1987; Levine 1983; Prüss 1983; Swart 1988 and others) observed periodic oscillations in age-structured models and it was believed that such periodic solutions may be induced by a Hopf bifurcation (Inaba 1998, 2002). However, there was no general Hopf bifurcation theorem available for age structured models at that time. Pierre and Shigui originally wanted to establish such a Hopf bifurcation theorem for general age structured models so that it can be used to analyze the existence of Hopf bifurcation for various types of age-structured models. It turned out that the project was much bigger than they expected: to establish a Hopf bifurcation theorem one needs to develop the center manifold theorem and normal form theory for general age structured models, which had not been investigated in the literature at that time.

Pierre and Shigui (and Zhihua Liu joined in later) then started to study this problem systematically. First, in their *Memoirs of Amer. Math. Soc.* (2009) paper Magal and Ruan (2009b) developed a center manifold theory for semilinear Cauchy problems with non-dense domain. Using the Liapunov-Perron method and following the techniques of Vanderbauwhede (1987), Vanderbauwhede (1989), Vanderbauwhede and van Gils (1987), Vanderbauwhede and Iooss (1989) in treating infinite dimensional systems, they established the existence and smoothness of center manifolds for semilinear Cauchy problems with nondense domain. The existence of center-unstable manifolds for nondensely defined Cauchy problems was considered in Liu et al. (2012). Then using the center manifold theorem associated with the integrated semigroup theory, they (Liu et al. 2014) developed a normal form theory for semilinear Cauchy problems in which the linear operator is not densely defined and is not a Hille-Yosida operator and presented procedures to compute the Taylor’s expansion and normal form of the reduced system restricted on the center manifold. Next they established in Liu et al. (2011) a Hopf bifurcation theorem for abstract Cauchy problems in which the linear operator is not densely defined and is not a Hille–Yosida operator. As applications, the main theorem was used to obtain a general Hopf bifurcation theorem for age-structured models.

Pierre and his coauthors as well as other researchers have applied these theories to study bifurcations and normal forms in various structured models, including age-structured models (Chu et al. 2016; Ducrot et al. 2023; Fu et al. 2015; Liu et al. 2016, 2015), size-structured models (Chu et al. 2009; Chu and Magal 2013), maturity structured models (Chu et al. 2011), an evolutionary epidemiological model of influenza A drift (Magal and Ruan 2010), a blood-stage malaria infection model (Su et al. 2011), the second-order semi-linear Gurtin–MacCamy equation (Ducrot et al. 2023), predator–prey models with age structure (Liu et al. 2021, 2016; Wu et al. 2024), etc.

### Other related studies

Pierre and his coauthors investigated other properties of solutions to abstract Cauchy problems with nondense domain, such as the essential growth rate for bounded linear perturbations (Ducrot et al. 2008), singular perturbations (Ducrot et al. 2011, 2016b, 2017; Magal and Seydi 2019), variation of constants formula and exponential dichotomy (Ducrot et al. 2015, 2016a; Magal and Seydi 2021), monotonicity (Magal et al. 2019), positively invariant subsets (Magal et al. 2021), etc. These studies enhance the theories of abstract Cauchy problems with nondense domain and will inspire further investigation on such equations.

Pierre and his coauthors also employed the abstract settings to study some other types of equations, including retarded functional differential equations (Liu et al. 2008), neutral functional differential equations (Ducrot et al. 2010), parabolic equations (Ducrot and Magal 2020; Ducrot et al. 2010), partial differential equations with time delay (Ducrot et al. 2013), age-structured models with diffusion (Ducrot et al. 2021), and so on. Using similar techniques, Pierre (Magal 2001a) examined the existence of compact attractors for time periodic age-structured population models and Pierre and Horst Thieme (Magal and Thieme 2004) studied the eventual compactness for semiflows generated by nonlinear age-structured models.

### Magal and Ruan’s 2018 monograph

In 2018, Pierre and Shigui published their monograph, *Theory and Applications of Abstract Semilinear Cauchy Problems*, Applied Mathematical Sciences **201**, Springer, New York, pp. 543. This monograph provides a self-contained and comprehensive presentation of the fundamental theory of non-densely defined semilinear Cauchy problems and their applications. D. Bugajewski (*zbMATH*, 2020) wrote that “This interesting monograph can be a useful tool for researchers interested in the theory of abstract differential equations along with their applications, especially in age-structured models. However, it can be also used by graduate students as well as PhD students who are willing to get familiar with this theory. $\ldots$ Remarks and Notes appearing at the end of each chapter are a good hint for further reading.” P. Georgescu (*Math. Reviews*, 2019) commented that “This book will be of great interest for researchers studying abstract ODEs and their applications, especially for those with interest in nonlinear population dynamics, particularly in age-structured models.”

## Traveling waves in biological systems

### Reaction-diffusion systems

The spatial spread of newly introduced species or diseases is a subject of continuing interest to both theoreticians and empiricists. Mathematically this is characterized as the existence of traveling waves in ecological or epidemic models described by reaction-diffusion systems. With initial conditions corresponding to a spatially localized introduction, such models predict the eventual establishment of a well-defined invasion front, which divides the invaded and uninvaded regions and moves into the uninvaded region with constant velocity (Murray 2003). Differing from competitive or cooperative population models, to establish the existence of traveling waves in susceptible-infectious (SI) models (Hosono and Ilyas 1995; Ruan 2007; Ruan and Wu 2009) and predator–prey models (Dunbar 1983, 1986; Huang et al. 2003) is more challenging due to the lack of monotonicity in such systems. Pierre and collaborators contributed in this classical topic. For a spatially structured SI epidemic model with vertical transmission, a logistic effect on vital dynamics and a density dependent incidence, Ducrot et al. (2012) first showed global stability of the endemic state in a bounded spatial domain, and then proved the existence of travelling wave solutions connecting the endemic and the disease-free states. Based on a detailed study of the center-unstable manifold around the interior equilibrium, they (Ducrot et al. 2013) further proved the existence of an infinite number of traveling wave solutions connecting the trivial equilibrium and the interior equilibrium. For the diffusive Rosenzweig-MacArthur predator–prey model (i.e., the predator–prey model with Holling type II function, see Huang et al. (2003)), Ducrot et al. (2021) studied the existence and uniqueness of a traveling wave connecting two equilibria or connecting an equilibrium point and a periodic wave train by coupling the theories of invariant manifolds together with those of global attractors.

Ducrot et al. (2018) studied the stability and bifurcation properties of the positive interior equilibrium for a reaction-diffusion equation with nonlocal advection. Under a general assumption on the nonlocal kernel they first established the local well posedness of the problem in suitable fractional spaces and obtained stability results for the homogeneous steady-state. As a special case, they showed that “standard” kernels such as Gaussian, Cauchy, Laplace and triangle, will lead to stability. Then they specified the model with a given step function kernel and investigated two types of bifurcations, namely Turing bifurcation and Turing-Hopf bifurcation. In fact, they proved that a single scalar equation may display this two types of bifurcations with the dominant wave number being sufficiently large.

### Age-structured models with diffusion

Notice that in population dynamics, species need to be mature enough to disperse. In transmission dynamics of infectious diseases, the infection age of the moving hosts is crucial for the spatial spread of the disease. So age-structured biological models with diffusion appear very naturally (Gurtin and MacCamy 1981; Hastings 1992; Langlais 1985; Webb 1980). Then the existence of traveling waves in age-structured ecological and epidemic models with diffusion becomes a very interesting and challenging problem. Working with Arnaud Ducrot and Shigui, Pierre made some fundamental contributions on this topic. For instance, Ducrot and Magal (2009) studied the existence of traveling waves for a class of epidemic models structured in space and with respect to the age of infection, and obtained a necessary and sufficient condition for the existence of traveling waves for such a class of problems. They (Ducrot and Magal 2011) further considered the existence of traveling waves for an infection-age structured epidemic model with constant recruitment and described the minimal wave speed. In Ducrot and Magal (2019), they constructed a smooth center manifold for a general class of abstract second order semilinear differential equations involving nondensely defined operators and applied the results to study periodic wave trains for the so-called Gurtin–McCamy equations; i.e. nonlocal age-structured models with diffusion. Ducrot et al. (2010) investigated the existence of traveling wave solutions in an age-structured model describing the spread of an infectious disease within a population of multigroups. The results apply to the crisscross transmission of feline immunodeficiency virus among cats and some sexual transmission diseases in multiple host groups.

### Keller-Segel equations

Based on Brownian motion and characterized by the diffusion coefficient, reaction-diffusion equations are standard to model interactions and movement of individuals from cells to vertebrate populations. Taking into account the individual movement behavior such as speed, run times, and turn angle distributions, reaction-transport equations arise naturally in modeling biological problems (Hillen and Hadeler 2005). The Keller-Segel model describes the directed movement of cells and organisms in response to chemical gradients, chemotaxis, and has attracted significant interest due to its applications in a wide range of biological phenomena (Hillen and Painter 2009). Traveling waves in Keller-Segel models are often observed in chemotactic aggregation phenomena like cell movement in response to chemical gradients.

Ducrot et al. (2011) analyzed a population dynamics model of a proliferating cell culture in *in vitro* settings with respect to spatial movement, cell-cell interaction, cell cycle phase, and proliferative status of individual cells in the culture. More specifically, they considered a monolayer of cells occupying a two-dimensional geometry, proliferating over a time course from an initial seeding to confluence and incorporated cell heterogeneity in the population by tracking the size of individual cells, which is correlated to the cell cycle. They also incorporated proliferative status by tracking transition to and from quiescence of individual cells in the population.

Fu et al. (2020) discussed a cell-cell repulsion model based on a hyperbolic Keller-Segel equation with two populations, which aims at describing the cell growth and dispersion. From a numerical perspective, they observed that their model admits a competitive exclusion principle, which is different from the classical competitive exclusion principle for the corresponding ODE model, and showed that the impact of the initial distribution on the proportion of each species in the final population lies in the initial number of cell clusters and that the final proportion of each species is not influenced by the precise distribution of the initial distribution. They also found that a fast dispersion rate gives a short-term advantage while the vital dynamics contributes to a long-term population advantage. Fu et al. (2021a) described a hyperbolic model with cell-cell repulsion with a dynamics in the population of cells; that is, a population of cells produces a field which induces a motion of the cells following the opposite of the gradient. The field indicates the local density of population and it is assumed that cells try to avoid crowded areas and prefer locally empty spaces which are far away from the carrying capacity. They analyzed the well-posed property of the associated Cauchy problem on the real line and obtained a convergence result for bounded initial distributions which are positive and stay away from zero uniformly on the real line. Fu et al. (2021b) and Griette et al. (2025) investigated the existence of sharp discontinuous and continuous traveling waves, respectively, in a hyperbolic Keller-Segel equation for a population of cells producing a field which induces a motion of the cells following the opposite of the gradient.

## Uniform persistence and global attractors

Uniform persistence is an important concept in population dynamics since it characterizes the long-term survival of some or all interacting species in a biological system. From the point of view of dynamical system theory, uniform persistence means that a closed subset of the state space (e.g., the set of extinction for one or more populations) is repelling for the semiflows on the complementary set (i.e., on the boundary). A natural question is about the existence of “interior” global attractors and “coexistent” steady states for uniformly persistent (permanent) dynamical systems. The issue was addressed by Hale and Waltman (1989), Hutson and Schmitt (1992), etc. In Magal and Zhao (2005), by appealing to the theory of global attractors on complete metric spaces, Pierre and Xiao-Qiang Zhao obtained weaker sufficient conditions for the existence of interior global attractors for uniformly persistent dynamical systems, and hence generalized the earlier results on coexistent steady states. Their paper is now a highly-cited article since their results are very useful to prove the global stability of coexistence steady states in structured biological models (see Magal et al. 2010; Ma and Magal 2024).

The perturbation of a globally stable steady state was considered by Smith and Waltman (1999). Magal, (2009) used the upper semi-continuity of a parametrized family of global attractors and investigated the case where the linearized equation of the unperturbed system has a simple dominant eigenvalue 0 in the case of a continuous time system (or 1 in the case of a discrete time system), so that such a system may exhibit a bifurcation. Pierre also described the global dynamics of the perturbed system.

## Transfer problems in population dynamics

Pierre and his collaborators investigated transfer problems in population dynamics. Here we introduce their studies on two topics: cell-to-cell P-glycoprotein transfer in breast cancers and economic transfers in a population.

### Cell-to-cell P-glycoprotein transfer in breast cancers

The efflux protein P-glycoprotein (P-gp) is overexpressed in many cancer cells and has known capacity to confer multidrug resistance to cytotoxic therapies and cell-to-cell P-gp transfers have been observed. Pierre and his collaborators (Pasquier et al. 2011) reported cell-to-cell transfers of functional P-gp in co-cultures of a P-gp overexpressing human breast cancer MCF-7 cell variant, selected for its resistance towards doxorubicin, with the parental sensitive cell line. They found that P-gp as well as efflux activity distribution are progressively reorganized over time in co-cultures analyzed by flow cytometry. They then constructed a mathematical model based on a Boltzmann type integro-partial differential equation structured by a continuum variable corresponding to P-gp activity that describes the cell populations in co-culture. The mathematical model elucidates the population elements in the experimental data, specifically, the initial proportions, the proliferative growth rates, and the transfer rates of P-gp in the sensitive and resistant subpopulations. Later they (Pasquier et al. 2012) identified intercellular membrane nanotubes, also termed cytonemes or tunneling nanotubes (TnTs), as short distance P-gp carriers between MCF-7 cells in vitro. Their results indicate that local P-gp transfers mediated by TnTs co-exist with remote P-gp transfers via microparticles and are together responsible for the extragenetic emergence of multi-drug resistance in a sensitive subpopulation co-cultured with resistant cells. Assuming that cells continually encounter each other, and from some hypotheses on cell-to-cell rules of transfer, they (Magal et al. 2017) further derived discrete and continuous Boltzmann-like integrodifferential equations which were used to fit the experimental data of cell-to-cell protein transfer in breast cancer. They (Hinow et al. 2009) generalized the model by introducing proliferation of individuals and production and diffusion of the transferable quantity and showed that the generalized model admits a globally asymptotically stable steady state, provided that transfer is sufficiently small. They (Dyson et al. 2019) also provided a mathematical model for the direct cell-to-cell transfer of proteins between cells and the indirect transfer between cells and the surrounding liquid. After analyzing the model and presenting some numerical simulations, they observed that the fit with the experimental data was better when the indirect transfer of the protein released in a dish was taken into account.

### Economic transfers in a population: Robin Hood model vs Sheriff of Nottingham model

Griette and Magal (2025) investigated the transfer problem in a population structured by a continuous variable corresponding to the quantity being transferred and proposed a model in the form of an integro-differential equation with kernels corresponding to the specific rules of the transfer process. After establishing the well-posedness of the Cauchy problem in the space of measures, they characterized transfer kernels that give a continuous semiflow in the space of measures and derivedd a necessary and sufficient condition for the stability in the space $L^1$ of integrable functions. They constructed some examples of kernels that may be particularly interesting in economic applications and considered transfers of economic value (e.g., money) between individuals. The two models are the “Robin Hood model,” where the richest individual unconditionally gives a fraction of their wealth to the poorest when a transfer occurs, and the “Sheriff of Nottingham model,” where the richest unconditionally takes a fraction of the poorest’s wealth. Between these two extreme cases there is a continuum of intermediate models obtained by interpolating the kernels. Finally they illustrated those models with numerical simulations and showed that any small fraction of the “Sheriff of Nottingham” in the transfer rules leads to a segregated population with extremely poor and extremely rich individuals after some time.

Magal and Webb (2000) and Magal (2002) introduced a model devoted to transfers of genetic material in which parent cells exchange genetic material to form their offspring. For more recent studies on structured population models with transfers, we refer to Magal and Raoul (2015, 2025).

## Modeling of epidemic problems

Pierre worked extensively on modeling various epidemic problems of high relevance to society. This includes the increased concern about infections with antibiotic-resistant bacteria, *Salmonella* infections, vector-borne diseases, and viral diseases such as COVID-19.

### Infections of antibiotic-resistant bacteria

Antimicrobial resistance has been described as one of the biggest threats to human health in the 21st century. Antibiotic-resistant bacteria (ARB), found in people, animals, plants, and the environment, have rapidly emerged and spread throughout the world, threatening our ability to treat infectious diseases, resulting in prolonged illness, disability, and death, and increasing the cost of health care. In 2000s, the collaborating team of Erikia D’Agata (Harvard Medical School at that time), Glenn, Pierre and Shigui made great effort and important contributions in modeling the infection and spread of ARB in hospital settings as well as in the community. In their 2005 PNAS paper (Webb et al. 2005), they formulated a coupled ODE-PDE two-level population model to quantify key elements in nosocomial (hospital-acquired) infections. At the bacteria level, patients infected with these strains generate both nonresistant and resistant bacteria. At the patient level, susceptible patients are infected by infected patients at rates proportional to the total bacteria load of each strain present in the hospital. The objectives were to analyze the dynamic elements of nonresistant and resistant bacteria strains in epidemic populations in hospital environments and to provide understanding of measures to avoid the endemicity of resistant antibiotic strains. Their coupled ODE-PDE modeling scheme has now become standard in modeling two-scale (such as within-host and between-host, immuno-epidemiological) infectious diseases (Ghosh et al. 2023; Li et al. 2021). The mathematical analysis of a reduced submodel (at the bacteria level and patient level with only one strain) was carried out in D’Agata et al. (2006). This reduced model can also be regarded as a susceptible-infectious epidemic model with infection-age. Magal et al. (2010) studied, among other properties, the global asymptotical stability of the unique endemic equilibrium of this reduced model by constructing a Liapunov functional. Interestingly, their technique of choosing Liapunov functionals has been followed by many researchers for treating similar age-structured models and their work is now a very highly-cited paper. Such models were also generalized by Pierre and collaborators to include multiple groups, see (Magal and McCluskey 2013; Magal et al. 2016, 2018).

Considering the fact that the populations in hospital wards are small, the team plus Damien Olivier (D’Agata et al. 2006) developed a comprehensive individual-based model (IBM) to describe the complex transmission dynamics of antimicrobial-resistant bacteria in hospitals. They simplified the interpretation of the IBM by developing a corresponding deterministic model (DEM), which describes the average behavior of the IBM over a large number of simulations. The integration of these two model systems provides a quantitative analysis of the emergence and spread of antibiotic-resistant bacteria, and demonstrates that early initiation of treatment and minimization of its duration mitigates antibiotic resistance epidemics in hospitals. In D’Agata et al. (2008), incorporating several key factors contributing to the emergence and spread of resistant bacteria including the effects of the immune system, acquisition of resistance genes and antimicrobial exposure, they created a model to identify key strategies which would limit the emergence of antimicrobial-resistant bacterial strains. Their simulations show that early initiation of antimicrobial therapy and combination therapy with two antibiotics prevent the emergence of resistant bacteria, whereas shorter courses of therapy and sequential administration of antibiotics promote the emergence of resistant strains. These findings provide new insights into strategies aimed at optimizing the administration of antimicrobials for the treatment of infections and the prevention of the emergence of antimicrobial resistance.

### *Salmonella* Infection

*Salmonella* is one of the major sources of toxi-infection in humans. The incidence of human salmonellosis has increased greatly in recent years and this can mostly be attributed to epidemics of *Salmonella enteritidis* in poultry in many countries and the association between egg consumption and *S. enteritidis* outbreaks is a serious international economic and public health problem. Pierre and his coauthors (Prevost et al. 2006) formulated a model for *S. enteritidis* transmission in hen houses, taking account both the hens and the environmental bacterium contamination. By considering the hen’s individual development of the disease, they built a model for the production of eggs contaminated by *S. enteritidis* and provided understanding of measures to avoid the endemicity of *S. enteritidis* in industrial hen houses. In Prevost et al. (2007), they presented a model incorporating a spatial structure for the dynamics of *Salmonella* infection within an egg laying hen flock and investigated the existence and uniqueness of solutions for the spatially structured model. Based on data of a selection experiment for increased or decreased rate of carrier-state and using the model in Prevost et al. (2006), they (Prévost et al. 2008) further showed that mixing, in an equal proportion, animals issued from a line selected for a lower or higher propensity to carry *Salmonella*, results in a reduction by half of the maximal percentage of contaminated animals but does not accelerate the extinction of the disease. These results show the importance of introduction of animals selected for a reduced rate of *Salmonella* carrier-state within commercial flocks, even at a rather moderate percentage. In Zongo et al. (2010), they proposed an individual-based model which describes the spatio-temporal spread of *Salmonella* within a laying flock and takes into account the host response to bacterial infection. The model allows studying the effects, on the spatiotemporal distribution of the bacteria, of both mean and variability of different elements of host response. Finally, they (Beaumont et al. 2012) presented a review of data available on the possibility of reduction of fowl carrier-state through genetic selection. It would enhance the benefits that may be obtained from an integrated approach of genetics of resistance in hens to improve the control of the incidence of *Salmonella* within a flock.

### Epidemic models with spatial structure

Magal et al. (2018) considered a reaction–diffusion vector-host epidemic model and studied the existence and global stability of an endemic equilibrium by combining arguments from monotone dynamical system theory, persistence theory, and the theory of asymptotically autonomous semiflows. They (Magal et al. 2019) further defined the basic reproduction number $R_0$ for the reaction-diffusion epidemic models as the spectral radius of a product of a local basic reproduction number *R* and strongly positive compact linear operators with spectral radii one, and investigated $R_0$ for the reaction–diffusion vector-host epidemic model. In Magal et al. (2020), they developed a reaction-diffusion equation model for the spatial spread of an infectious disease in a geographical setting and applied it to simulate the spatial spread of the 2016–2017 seasonal influenza epidemic in Puerto Rico. Ducrot and Magal (2022) proposed a mathematical model to describe the local movement of individuals by taking into account their return to home after a period of travel and applied the return to home process to an epidemic. In light of the COVID-19 pandemic, human travel is critical to spread the virus at the scale of a city, a country, and the scale of the earth. Pierre (Magal 2024) improved the previous model in Ducrot and Magal (2022) by introducing a third compartment composed of immobile individuals consisting mostly of office workers, and obtained a convergence result of solutions to an equilibrium distribution by using semigroup theory.

### SIR Models: contacts between individuals and unreported cases

*(a) Modeling contacts between individuals*. The classical susceptible-infectious-recovered (SIR) model, originated from the seminal papers of Ross (1916) and Ross and Hudson (1917a, 1917b), and the fundamental contributions of Kermack and McKendrick (1927, 1932, 1933); Kermack (1937, 1939), describes the transmission of infectious diseases between susceptible and infective individuals and provides the basic framework for almost all later epidemic models, including stochastic epidemic models using Monte Carlo simulations or Individual-Based Models (IBM). In Magal and Ruan (2014), by defining the rules of contacts between susceptible and infective individuals, the rules of transmission of diseases through these contacts, and the time of transmission during contacts, Pierre and Shigui provided detailed comparisons between the classical deterministic SIR model and the IBM stochastic simulations of the model. More specifically, for the purpose of numerical and stochastic simulations they distinguished two types of transmission processes: that initiated by susceptible individuals and that driven by infective individuals. Their analyses and simulations demonstrate that in both cases the IBM converges to the classical SIR model only in some particular situations. In general, the classical and individual- based SIR models are significantly different. Their study reveals that the timing of transmission in a contact at the individual level plays a crucial role in determining the transmission dynamics of an infectious disease at the population level. Their results are particularity important and useful for epidemics that show a large fraction of unreported cases, such as in COVID-19 outbreaks.

*(b) Identifying unreported cases*. During outbreaks of infectious diseases, in particular those newly emerging infectious diseases, there were a large fraction of cases that were not identified and thus were not reported. In many cases the ratio of unreported to reported cases is very high. This would affect the understanding of the disease and impact the implementation of measures for controlling the epidemic. Based on the standard SIR model, Pierre and Glenn (Magal and Webb 2018) introduced two new compartments, *CR*(*t*) and *CU*(*t*), the cumulative number of reported and unreported cases at time *t* respectively, and derived an equation for *CR*(*t*). They estimated the ratio of unreported to reported cases by the identification of parameters for the model from reported case data. Later Pierre used this technique to handle COVID-19 data, see next subsection.

### Data-driven mathematical modeling approaches for COVID-19

During the COVID-19 pandemic, mathematical modeling played a crucial role in understanding the infection, mutation, prevention, control, and vaccination of the new virus SARS-CoV-2 and its rapid global spread. Since Pierre traveled to China in late 2019 and visited Wuhan when COVID-19 outbreak began there, he immediately started to model the disease after returning to France. Collaborating with Zhihua Liu, Ousmane Seydi and Glenn, their first paper on modeling the COVID-19 outbreak in Wuhan (Liu et al. 2020) was submitted as early as on February 2, 2020 and was published on March 8, 2020. Subsequently, he and his coauthors published more than a dozen modeling papers on various aspects of COVID-19 transmission, from latent period, unidentified cases, parametric identification, testing data, vaccine efficacy to real-time prediction. Their techniques and results were later summarized in a long and detailed review by Jacques and Pierre (Demongeot et al. 2024). In order to invite researchers around the world to present multiple aspects of mathematical modeling to address important problems relevant to infectious disease (in particular COVID-19) outbreaks, Pierre also organized the Europe-North America and Europe-Asia Infectious Disease Outbreaks webinar series from October 2020 to June 2021 and from October 2021 to June 2022, respectively, which are stored on YouTube.^1^

The following techniques were first reported in Demongeot et al. (2020) and were finalized in Demongeot et al. (2024).

Two types of models have been applied to study the transmission dynamics of infectious diseases. The first are the so-called phenomenological models, originated from Lambert (1758) and Euler (1767), which are used to reproduce the data and their tendency with a limited number of parameters. The second are the mechanistic epidemic models, initiated by Bernoulli (1760) and d’Alembert (1761), which consists of differential equations describing the evolution of infectious diseases. The basic epidemic model involves the numbers of susceptible individuals *S*(*t*) and infectious individuals *I*(*t*) at time *t* of a population and is governed by

7.1 $$\begin{aligned} \begin{aligned}&S'(t)=- \tau (t) S(t)I(t), \\&I'(t)=\tau (t) S(t)I(t) -\nu I(t) \end{aligned} \end{aligned}$$

with initial values $S(t_0)=s_0\ge 0, I(t_0)=I_0\ge 0$ at the initial time $t_0.$ The function $\tau (t)$ is the transmission rate and the constant $\nu$ is the reciprocal of the average duration of the asymptomatic infectious period.

At the initial stage of an epidemic, the number of susceptible individuals and the transmission rate can be supposed to be constant as $S_0$ and $\tau _0$, respectively, so the number of infectious individuals increases exponentially as $I(t)=I_0 e^{\chi _2 (t-t_0)},$ in which $\chi _2=\tau _0 S_0-\nu ,$ and $CR(t)=\chi _1(e^{\chi _2 (t-t_0)} -1) + \chi _3,$ where *CR*(*t*) denotes the cumulative number of reported cases at time *t*, $\chi _1$ and $\chi _3$ are evaluated from the data which in turn yield the values for $I_0$ and $t_0$ (Demongeot et al. 2024; Magal and Webb 2018). After the initial stage, the data and the exponential growth diverge and the transmission rate is no longer a constant. To understand and monitor the epidemiology of the infectious disease, estimating the average transmission rate becomes the most crucial challenge, which is the combination of three factors: (i) the coefficient of virulence of the infectious agent may change over time due to mutation over the course of the disease history; (ii) the coefficient of susceptibility of the host may also change if mitigation measure have been taken; and (iii) the number of contacts per time unit between individuals (see Magal and Ruan 2014) changes due to personal activities and changed social behavior.

Notice that the transmission rate $\tau (t)=\frac{\textrm{the probability of transmission}}{\textrm{the average duration of contact}}$ changes along time since people try to protect themselves and the number of contacts per unit of time reduces gradually. To find an explicit formula for $\tau (t),$ consider the Bernoulli-Verhulst equation for *CR*(*t*) : 

7.2 $$\begin{aligned} CR'(t)=\chi _2 CR(t) \left[ 1 - \left( \frac{CR(t)}{CR_{\infty }}\right) ^{\theta }\right] , \;\; t\ge t_0; \;\; CR(t_0)=CR_0\ge 0, \end{aligned}$$

where $\chi _2, \theta , CR_{\infty }$ are parameters and $CR_0$ is the initial value, all of them can be evaluated from data. The solution of (7.2) is given by

7.3 $$\begin{aligned} CR(t)=\frac{e^{\chi _2 (t-t_0) CR_0}}{\left[ 1+\frac{CR_0^\theta }{CR_{\infty }^\theta } (e^{\chi _2 (t-t_0)}-1)\right] ^{\frac{1}{\theta }}}. \end{aligned}$$

Hence, $\tau (t)$ can be expressed as

7.4 $$\begin{aligned} \tau (t)=\frac{\nu f \left\{ \chi _2 \left[ 1-(1+\theta ) \left( \frac{CR(t)}{CR_{\infty }}\right) ^{\theta }\right] +\nu \right\} }{\nu f(I_0+S_0)+\nu CR_0 -CR(t)\left\{ \chi _2\left[ 1-\left( \frac{CR(t)}{CR_{\infty }}\right) ^{\theta }\right] +\nu \right\} }. \end{aligned}$$

Formulas (7.3) and (7.4) provide an explicit formula for the transmission rate $\tau (t)$ which is a phenomenological model with a few parameters. Pierre and his collaborators used the phenomenological model to calibrate COVID-19 data from different countries and regions, including Brazil, China, France, Germany, Italy, South Korea, Spain, United Kingdom, California and New York. See the review (Demongeot et al. 2024) and references cited therein. The model was also modified to characterize multiple epidemic waves and fit the cumulative reported COVID-19 data from France. The transmission rate $\tau (t)$ was applied to calculate the instantaneous reproduction number $R_e(t)=\tau (t)S(t)/\nu$ and the quasi-instantaneous reproduction number $R^0_e(t)=\tau (t)S_0/\nu ,$ which can be used to determine whether the epidemic persists or dies out in the long term. COVID-19 epidemic models with more compartments and age groups were also considered. Their methods and techniques, despite their mathematical rigor and timely applicability, are valuable tools in linking differential equation models in biology and epidemiology with modern methods of data fitting, allowing a good understanding of factors that influence fluctuations in time-dependent transmission rates.

Pierre and his coauthors (Demongeot et al. 2023) studied the parameter identifiability of the Kermack-McKendrick model with age of infection which takes into account this dependency. By considering a single cohort of individuals, they showed that the daily reproduction number can be obtained by solving a Volterra integral equation that depends on the flow of new infected individuals. They applied the method to a dataset from SARS-CoV-1 with detailed information on a single cluster of patients and emphasized the necessity of taking into account the initial data in the analysis to ensure the identifiability of the problem.

## Two volumes of “Differential Equations and Population Dynamics”

In 2022–25, Ducrot, Griette, Liu and Pierre published a comprehensive monograph on “Differential Equations and Population Dynamics”, which consists of two volumes. Volume I contains “Introductory Approaches” (Ducrot et al. 2022) and Volume II “Advanced Approaches” (Ducrot et al. 2025). Volume I presents the basic theoretical concepts of dynamical systems with applications in population dynamics. It contains topics such as existence, uniqueness and stability of solutions, global attractors, bifurcations, center manifold and normal form theories, as well as cutting-edge applications, including a Holling’s predator–prey model with handling and searching predators and projecting the epidemic forward with varying level of public health interventions for COVID-19. Volume II introduces new perspectives on predator–prey systems by applying theoretical results to derive oscillating solutions through Hopf bifurcation, traveling invasion waves using global attractor theory, and a description of long-term dynamics in competitive interactions between predator variants. Throughout the two volumes, concepts are illustrated with numerical examples and MATLAB codes are provided.

These two volumes bridge the gap between mathematics, biology and medicine by integrating relevant concepts from these subject areas, making them self-sufficient for the reader. It will be a valuable resource to graduate and advanced undergraduate students for interdisciplinary research in the area of mathematics and population dynamics.

## Final words

Pierre’s life ended suddenly when he just shortly passed his 56th birthday, almost the same age when his Ph.D. thesis supervisor Ovide Arino passed away. The differential equations and mathematical biology community once again lost an active and outstanding researcher, a passionate leader, and a considerate mentor. To those who did not know him well, Pierre sometimes sounded impatient and blunt when he was talking about mathematics, but that was mainly because of his high expectation and straightforwardness, from his heart he was friendly and very willing to help others. He cared very much about his students and visitors, in particular those from developing countries. Pierre never liked to follow rules, but he was very honest, very collaborative, and very enthusiastic, and the fact that he had more than 70 collaborators simply demonstrates that. He also was concerned about social inequality and showed great sympathy to the poor people in France and worldwide. On top of his research, teaching and professional activities, Pierre played guitar and owned quite a few good ones. He enjoyed traveling around the world, and hiking and camping in wilderness areas. Pierre was a very good cook and his plan was to open a French restaurant in China when he retires.

Pierre, with a smart mind, a kind character and an interesting soul, will be missed by his friends, colleagues and students. His work will have lasting value for researchers in the theoretical and applied aspects of the subjects he studied and his techniques, methods, and ideas will be carried over by young and future generations.

## Data Availability

No data was used in this study.

## Footnotes

1 https://www.youtube.com/@bordeauximbinfectiousdisea3512https://www.youtube.com/@ledzeeeeeeeeeeep
